# Clinical Neuropathology practice guide 6-2013: morphology and an appropriate immunohistochemical screening panel aid in the identification of synovial sarcoma by neuropathologists 

**DOI:** 10.5414/NP300685

**Published:** 2013-10-16

**Authors:** Julia Lee Keith, Juan Bilbao, Sidney Croul, Lee Cyn Ang, Marie-Christine Guiot, John Rossiter, Zeina Ghorab, Cynthia Hawkins, Jason Karamchandani

**Affiliations:** 1 Sunnybrook Health Sciences Centre, Department of Anatomical Pathology,; 2 University Health Network, Department of Pathology, University of Toronto, Toronto,; 3 London Health Sciences Centre, University Hospital, University of Western Ontario, London, Ontario,; 4 Montreal Neurological Hospital and Institute, McGill University Hospital Centre, Department of Pathology, University, Montreal, Quebec,; 5 Kingston General Hospital, Department of Pathology and Molecular Medicine, Queens University, Kingston, Ontario,; 6 Hospital for SickKids, Department of Pediatric Laboratory Medicine, and; 7 St. Michael’s Hospital, Department of Pathology, University of Toronto, Toronto, Ontario, Canada

**Keywords:** synovial sarcoma, nerve, nervous system, SOX10, BAF47, malignant peripheral nerve sheath tumor

## Abstract

Aims: Pathologists are under increasing pressure to accurately subclassify sarcomas, yet neuropathologists have limited collective experience with rare sarcoma types such as synovial sarcoma. We reviewed 9 synovial sarcomas affecting peripheral nerve diagnosed by neuropathologists and explored the morphologic and immunohistochemical differences between these and MPNST. Our goal was to make practical recommendations for neuropathologists regarding which spindle cell tumors affecting nerve should be sent for SYT-SSX testing. Methods: Clinical records and genetics were reviewed retrospectively and central pathology review of 9 synovial sarcomas and 6 MPNST included immunohistochemistry for SOX10, S100, BAF47, CK (lmw, pan, CK7, CK19), EMA, CD34, bcl2, CD99, and neurofilament. Results: Common synovial sarcoma sites were brachial plexus, spinal and femoral nerve, none were “intra-neural”, all had the SYT-SSX1 translocation, and 6/9 were monophasic with myxoid stroma and distinct collagen. Half of the monophasic synovial sarcomas expressed CK7, CK19 or panCK in a “rare positive cells pattern”, 8/9 (89%) expressed EMA, and all were SOX10 immunonegative with reduced but variable BAF47 expression. Conclusions: We recommend that upon encountering a cellular spindle cell tumor affecting nerve neuropathologists consider the following: 1) SYT-SSX testing should be performed on any case with morphology suspicious for monophasic synovial sarcoma including wiry or thick bands of collagen and relatively monomorphous nuclei; 2) neuropathologists should employ a screening immunohistochemical panel including one of CK7, panCK or CK19, plus EMA, S100 and SOX10, and 3) SYT-SSX testing should be performed on any spindle cell tumor with CK and/or EMA immunopositivity if SOX10 immunostaining is negative or only labels entrapped nerve elements.

## Introduction 

Synovial sarcoma is a malignant sarcoma usually arising in deep soft tissue of the extremities, defined by balanced t(X;18) chromosomal translocation, with 3 histologic patterns (monophasic, biphasic and poorly differentiated). The era of personalized histology-driven sarcoma therapy demands that pathologists accurately sub-classify spindle cell malignancies, but as synovial sarcoma rarely affects the nervous system neuropathologists’ collective experience with these tumors is somewhat limited. There is a relative paucity of literature regarding the neuropathology of synovial sarcoma, and this mostly describes rare intra-neural monophasic spindle cell tumors with a differential diagnosis of malignant peripheral nerve sheath tumor (MPNST). The present study reviewed 9 synovial sarcomas affecting peripheral nerve to an extent which necessitated resection by neurosurgery and consequent assessment by neuropathologists. Our aim was to provide recommendations for neuropathologists regarding which spindle cell tumors affecting nerve should be sent for t(X;18) testing: when presented with a cellular spindle cell tumor are there histologic clues to help distinguish between monophasic synovial sarcoma and MPNST? Which immunohistochemical markers should neuropathologists use to “screen” for a potential synovial sarcoma case? 

## Materials and methods 

After obtaining Research and Ethics Board approval, the pathology archives from 5 academic hospitals in Ontario and Quebec, Canada, were searched for a diagnosis of “synovial sarcoma”, and the operative, imaging, and pathology reports of these cases were reviewed. The authors selected those cases that were assessed by neuropathologists; these had mostly been removed by neurosurgeons and they all significantly affected nerves as described in the operative note and/or imaging studies (N of 12). Molecular analysis on these 12 tumors for t(X;18) SYT-SSX1 or SYT-SSX2 translocation via PCR confirmed the diagnosis of synovial sarcoma in 8 patients (1 patient had a classic biphasic morphology and immunohistochemistry so molecular testing was not deemed necessary), and these 9 synovial sarcoma cases are described in Tables 1 and Table 2. Three patients with an original histologic diagnosis of “synovial sarcoma” subsequently had molecular studies negative for both t(X;18) SYT-SSX1 and SYT-SSX2 translocation. These 3 “synovial sarcoma mimic” cases were reviewed by a pathologist with expertise in soft tissue pathology who felt that they were best classified as alternative diagnoses so they were excluded from the synovial sarcoma study group, however the immunohistochemical panel was applied for comparison purposes. The pathology archives from several hospitals were also searched for a diagnosis of “MPNST” and 6 cases which met diagnostic criteria for MPNST [1] were retrieved and reviewed. In all 6 of these MPNST cases a diagnosis of cellular schwannoma was excluded based on cellularity and proliferation [2]. 

The best paraffin blocks from each of the 9 synovial sarcoma cases, 6 MPNST and 3 synovial sarcoma mimics were retrieved and stained with H&E and immunohistochemistry for SOX10, S100, CK (low molecular weight (lmw), pan, 7 and 19), epithelial membrane antigen (EMA), CD34, bcl2, CD99, neurofilament (NF) and Ki67, the results of which were documented by a single neuropathologist (JLK) as negative or positive (+, ++ or +++). BAF47 staining was only scored if there was strong nuclear positivity of endothelial cells, and was considered “reduced” if at least 80% of tumor nuclei had weaker nuclear positivity than the endothelia. All immunohistochemistry was performed on the Ultra Benchmark automated stainer with Ultraview DAB Polymers Detection System and the antibodies listed in Table 1. 

## Results 

### Clinical features 

As shown in Table 2 the 9 synovial sarcoma patients were mostly women with an average age of 50 years (range 43 – 74) lacking relevant family history or previous radiation with primary tumors affecting the vertebrae/spinal nerves roots, brachial plexus, and femoral nerve (2 cases each). Most synovial sarcomas behaved aggressively with recurrences, 3 metastases occurred (all to lung), and 1 patient had 9 year recurrence free survival. 

### Histology 

**Figure 1:**
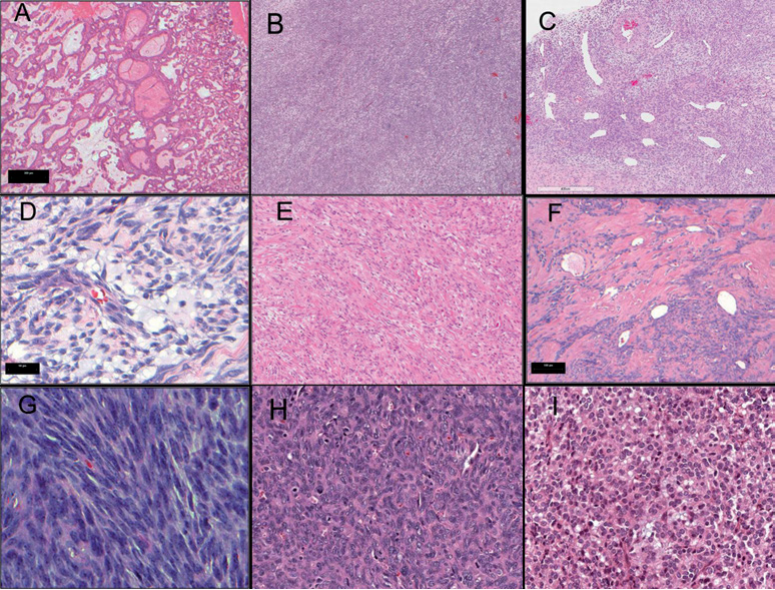
Figure 1. Histologic features of synovial sarcoma of the nervous system; A: biphasic architecture; B: fascicular fibrosarcoma-like growth pattern; C: dilated hemangiopericytoma-like vasculature and myxoid background; D: myxoid background; E: thin wiry collagen; F: thick collagenous bands; G: oval overlapping nuclei with small nucleoli; H: plump oval nuclei; I: round nuclei in a metastatic lesion.

As shown in Table 3 and Figure 1, 3/9 synovial sarcomas were biphasic with predominance of the glandular component comprised of papillae and irregular glands of various sizes lined by cuboidal cells (Figure 1A), 6/9 were monophasic with fascicular architecture (Figure 1B), all had dilated “hemangiopericytoma-like” vasculature (Figure 1C) and at least focal myxoid change (Figure 1C, D) which was extensively present in one case. 5/6 monophasic cases had either wiry collagen (Figure 1E) or thick bands of collagen (Figure 1F). There were no examples of the de-differentiated synovial sarcoma subtype. The nuclei within a given tumor were relatively monomorphous without pronounced atypia, and the nuclear morphology ranged between cases from elongated and overlapped with small nucleoli (Figure 1G), to plump oval nuclei (Figure 1H), and in one metastasis the nuclei were round (Figure 1I). The proliferative rate varied from 1 – 30 mitoses/10 hpf, and 5 cases had fewer than 4 mitoses/10 hpf, several of whom had relatively long disease free survival. In 3 synovial sarcoma cases candidate parent nerve was present at the edge of the tumor, and NF immunopositive axons were not distributed throughout any synovial sarcoma to support an endoneurial location. 

In comparison, the 6 MPNST contained neither the distinctive collagen nor myxoid stroma and the nuclei were more atypical, and the synovial sarcoma mimics were malignant spindle cell tumors with more severe nuclear atypia and fascicular architecture lacking both myxoid change and distinctive collagen. 

### Immunohistochemistry 

**Figure 2:**
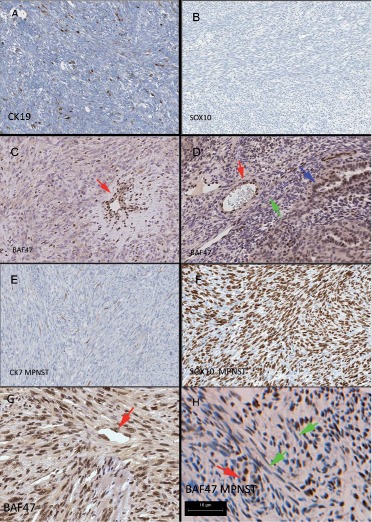
Figure 2. Immunohistochemistry of synovial sarcoma of the nervous system: A: common “rare positive cell” pattern of CK labeling in monophasic synovial sarcoma; B: all synovial sarcomas were SOX10 negative; C: reduced BAF47 staining of synovial sarcoma nuclei compared with endothelial nuclei (red arrow); D: biphasic synovial sarcoma showed strong nuclear staining of BAF47 of epithelial cells (blue arrow) comparable to endothelia (red arrow) while tumor stromal cells had reduced staining (green arrow). In comparison, the MPNST had: E: rare positive cells on CK staining; F: SOX10 positivity of varying numbers of nuclei, including cases with strong diffuse nuclear staining; G: BAF47 nuclear staining comparable to endothelial cells (red arrows); H: variable MPNST nuclear BAF47 immunolabeling including some negative tumor nuclei (green arrows) compared with strongly immunopositive endothelia (red arrow).

The immunohistochemistry for the 9 synovial sarcomas, 6 MPNST and 3 synovial sarcoma mimics is presented in Table 4 and Figure 2. 3/6 monophasic synovial sarcomas were positive for CK and 3 CK (CK7, CK19 and panCK) had comparable sensitivity (50% of monophasics, 67% overall) with a “rare scattered positive cells” pattern (Figure 2A). lmwCK was negative in all six monophasic synovial sarcomas, 3 of the monophasic synovial sarcomas were negative for all CK employed, and two of these CK negative cases had EMA expression (sensitivity of 83% for monophasics, 89% overall). 8/9 synovial sarcomas were S100 negative, with case 1 having focal S100 expression which was likely not entrapped parent nerve. All of the synovial sarcoma cases were SOX10 negative (Figure 2B). The 5 synovial sarcoma cases with interpretable results had reduced BAF47 staining (Figure 2C), variable BAF47 staining was noted within a given case, and no synovial sarcomas had complete absence of tumor nuclear staining. Within the biphasic synovial sarcomas there was strong BAF47 nuclear staining in endothelia and epithelial nuclei but the stromal component of the tumors had reduced BAF47 staining (Figure 2D). All synovial sarcomas were negative for CD34 and positive for both bcl2 and CD99. 

5/6 MPNST expressed S100 (83% sensitivity) and 3 MPNST had either CK or EMA staining, including the “rare positive cells” pattern (Figure 2E). One MPNST was S100 negative (Case 15). 4/6 MPNST were SOX10 positive ranging from strong diffuse nuclear staining (Figure 2F) to strong focal nuclear staining and the areas of focal SOX10 expression did not represent entrapped normal nerve elements by morphology or NF immunostaining. Two MPNST were SOX10 negative (including Case 11 (an epithelioid MPNST) and Case 15 (the possibility of synovial sarcoma was ruled out on genetic testing for SYT-SSX)) (67% sensitivity overall). 2/3 conventional MPNST had strong tumor nuclear staining with BAF47 (Figure 2G), but in one MPNST there was variable tumor nuclear staining including many nuclei of reduced staining intensity (Figure 2H). The epithelioid MPNST had reduced BAF47 immunolabeling. Two MPNST labeled with CD34 and all were positive for bcl2 and CD99. 

The 3 “synovial sarcoma mimics” were either CK negative or diffusely CK positive and none had the “scattered positive cells” pattern, two had some S100 expression, one had focal SOX10 immunolabeling. All 3 had strong tumor nuclear BAF47 staining and all expressed bcl2 and CD99. These 3 cases were assigned the following alternative diagnoses: malignant solitary fibrous tumor (Case 16), radiation induced sarcoma (MPNST vs. fibrosarcoma, Case 17), and spindle cell melanoma (Case 18). 

## Discussion 

Synovial sarcomas account for 10 – 15% of all soft tissue sarcomas and usually affect the deep soft tissue of the extremities of young adults [3]. The cell of origin is uncertain and despite their name they neither arise from nor differentiate towards synovium. They are defined by a balanced, reciprocal translocation involving fusion of the SYT gene at 18q11 to either SSX1 or SSX2 at Xp11 [3], with very rare cases having a fusion partner of SSX4 [4, 5, 6]. The 3 histologic subtypes are monophasic, biphasic and poorly differentiated. The SYT-SSX1 fusion occurs in ~ 2/3 of cases [7] and the SYT-SSX fusion type correlates with tumor histology with the majority of monophasic and biphasic synovial sarcomas harboring the SYT-SSX2 and SYT-SSX1 fusions respectively [8]. All of our cases had the SYT-SSX1 fusion, 1/3 of which had biphasic morphology. 

Histology-driven personalized therapy for soft tissue sarcoma is placing increasing pressure on all pathologists to accurately sub-classify these tumors [9]. This is especially prudent for synovial sarcoma given its relative chemosensitivity, especially to ifosfamide [10], and its directly tumorigenic SYT-SSX fusion protein which may one day provide a therapeutic target [11]. Monophasic synovial sarcoma may be difficult to recognize as they morphologically overlap with fibrosarcoma, hemangiopericytoma, cellular schwannoma and MPNST; the latter two are especially relevant when lesions involve nerves and/or present to neuropathologists vulnerable to a familiarity heuristic. The need for neuropathologists to correctly identify synovial sarcoma is reflected by Rodriguez et al. [12] recommended updates to the WHO classification of peripheral nerve tumors which add synovial sarcoma as the lone entry under “miscellaneous malignant intra-neural neoplasms”. 

Synovial sarcomas may present to neuropathologists in several different scenarios. Significant secondary involvement of a peripheral nerve by a typical soft tissue synovial sarcoma may require the involvement of neurosurgery thus invoking neuropathology; that this context applied to most cases in our series is not surprising, but the fact that neuropathologists are more likely to encounter synovial sarcomas that are not intra-neural has not been emphasized previously. Up to 5% of synovial sarcomas arise in the body axis with frequent involvement of the spinal nerve roots [13], as demonstrated by several of our cases. Synovial sarcoma rarely arises as a primary intra-neural tumor and the largest reported series has 10 patients [14]; none of our study cases were unequivocally endoneurial. Finally, there are isolated case reports of intra-cranial synovial sarcomas, either primary dural-based tumors [15, 16] or intra-cranial metastases from a systemic primary [17]. 

The literature is relatively sparse on synovial sarcomas affecting the nervous system, with the most significant contribution being Scheithauer et al. [14] 10 cases which were either primary intra-neural in origin or had endoneurial spread. Compared to their more common soft tissue counterparts the relatively unique features of Scheithauer’s intra-neural synovial sarcomas included over-representation of the monophasic subtype (90%) and SSX2 fusion partner [14]. Neither of these features was over-represented in our experience, likely because our series included synovial sarcomas secondarily affecting nerve and metastatic lesions. 

We sought to identify reliable histologic differences between synovial sarcoma and MPNST, and found the presence of wiry and thick bands of collagen and myxoid change to be clues to a synovial sarcoma diagnosis, and we were reminded that synovial sarcoma warrants consideration in the differential diagnosis for spindle cell tumors with less nuclear atypia and proliferation such as cellular schwannoma. Previous authors have described small overlapping or closely packed nuclei [1, 3], clusters of epithelioid cells highlighted by reticulin [3], wiry collagen [14] and/or collagenous bands [1], and stromal calcifications [1, 14] as features that supported a synovial sarcoma diagnosis over MPNST. 

How can neuropathologists best use immunohistochemistry to identify potential synovial sarcoma cases that warrant SYT-SSX testing? Focal CK or EMA expression is present in 90% of synovial sarcoma [3], and Scheithauer’s intra-neural synovial sarcomas had patchy expression of CK7, panCK and EMA in 100%, 80% and 90%, respectively [14]. Our study showed the “scattered CK positive cells” pattern to be useful and the sensitivities of CK7, CK19 and panCK were comparable (50% of monophasic synovial sarcomas, 67% overall). We suggest that for screening purposes neuropathologists employ any one of these three CK with the important addition of EMA as 2/3 of our CK negative synovial sarcomas were EMA positive (89% sensitivity of EMA for synovial sarcoma). We caution readers against assuming any spindle cell tumor with epithelial immunolabeling to be synovial sarcoma, as 3/6 of our MPNST cases had CK and EMA expression, which is comparable to previous studies and may have been due to cross-reactivity with schwannian components [12], and one must be cognizant of MPNST subtypes (epithelial and perineurial) with an epithelial immunophenotype. 1/3 synovial sarcoma mimics also had CK expression, although this was strong and diffuse. 

S100 remains a widely used tool for demonstrating neural crest lineage in spindle cell neoplasms, but results can be misleading in this setting. Karamchandani et al. [18] described S100 expression in 5 non-neural crest origin sarcomas, and 12/79 of their synovial sarcomas were S100 positive (15%). Others reported 30% of synovial sarcomas were S100 positive [7], half of Scheithauer’s synovial sarcomas had some S100 expression [14], and 1/9 (11%) of our synovial sarcomas expressed S100. SOX10 is a transcription factor in neural crest formation and specification to schwannian and melanocytic lineages, which is starting to replace S100 as a more sensitive and specific marker for schwannoma and neurofibroma [19] in the present authors’ practices. Although it was originally described by Nonaka et al. [19] to be more sensitive than S100 in detecting MPNST (49% vs. 30%), it is emerging as a more specific but less sensitive marker than S100 for MPNST. Nonaka and other authors have described a nuclear SOX10 labeling pattern in MPNST comparable to ours, ranging from focal to diffuse [19, 20]. Karamchandani et al. [18] reported that of 78 MPNST studied 18 were positive for both S100 and SOX10 (23%), 13 (17%) were S100+/SOX10-, 3 (4%) were S100-/SOX10+, and 44 cases (56%) were negative for both (the sensitivity of S100 for detecting MPNST was 40% and the sensitivity of SOX10 was 27%). None of their 5 “S100 positive sarcomas” expressed SOX10, which led the authors to conclude that in the setting of a soft tissue neoplasm SOX10 is more specific for peripheral nerve sheath tumors than S100. 4/5 of our conventional MPNST labeled with SOX10, which is better sensitivity (80%) than in previous larger studies. All 79 of Karamchandani’s synovial sarcomas were negative for SOX10 immunohistochemistry [18], and the 15 synovial sarcomas studied by Nonaka were negative for SOX10 [19]. We caution readers that in truly intra-neural synovial sarcomas the entrapped nerve elements may label with SOX10. One of our synovial sarcoma mimics was SOX10 negative, and as this case also had CK expression (Case 16, a malignant SFT) it was reasonable to pursue SYT-SSX testing to rule out synovial sarcoma. Although of limited sensitivity, a positive SOX10 result strongly supports a diagnosis of MPNST over synovial sarcoma, and with its other diagnostic applications for neuropathology this is a practical and accessible tool for neuropathologists to employ. 

Two recent papers suggested that a “decreased but not absent” pattern of BAF47/INI1 nuclear labeling was useful in distinguishing synovial sarcoma from histologic mimics [21, 22]. All 5 of our synovial sarcomas with interpretable BAF47 labeling had reduced nuclear positivity compared to endothelial cells, all 3 of our synovial sarcoma mimics had staining comparable to endothelia, and one of our conventional MPNST cases had reduced nuclear staining. Although our series is relatively small, our experience contrasted with Arnold et al. [21] description of a “uniform reduction of BAF47 within a given case”. Instead the intensity of nuclear staining within a tumor varied considerably in our series, with some cases being more reminiscent of the “mosaic pattern” described in familial and sporadic schwannomatosis and neurofibromatosis associated schwannomas [23] (Figure 2H). Indeed we worry about the inter-rater reliability of the variable “reduced but not absent pattern” of BAF47, we acknowledge that reduced BAF47 immunolabeling has been recognized in a growing number of tumor types [24], and caution that synovial sarcomas can change their BAF47 status upon recurrence [21]. For now the present authors plan to limit our use of BAF47 immunohistochemistry to the identification of the completely negative cells of malignant atypical teratoid rhabdoid tumors. 

TLE1 is an additional sensitive immunohistochemical tool for identifying synovial sarcoma [25, 26] and all of Scheithauer’s intra-neural synovial sarcomas expressed TLE1 [14]. The sensitivity of TLE1 for synovial sarcoma has been challenged, however, and one group of researchers found that 30% of MPNSTs also labeled with TLE1 [27]. TLE1 was not employed in our study, as reflective of its infrequent and limited range of diagnostic applications the antibody was not available in any of the participating centers. 

Based on our experience with this series and reviewing the literature we recommend that upon encountering a cellular spindle cell tumor affecting nerve neuropathologists consider the following regarding SYT-SSX testing: 

1) SYT-SSX testing should be performed on any case with morphology suspicious for monophasic synovial sarcoma including wiry or thick bands of collagen and relatively monomorphous nuclei. 

2) Neuropathologists should employ a screening immunohistochemical panel including one of CK7, panCK or CK19, plus EMA, S100 and SOX10. 

3) SYT-SSX testing should be performed on any spindle cell tumor with CK and/or EMA immunopositivity if SOX10 immunostaining is negative or only labels entrapped nerve elements. 

Lesions lacking the characteristic morphologic features which are immunonegative for both SOX10 and epithelial markers remain problematic and likely need to be handled on a case by case basis with the support of soft tissue pathology experts, but we caution neuropathologists against relying heavily on either S100 or BAF47 immunophenotype in this scenario, and add that other markers not employed in our study, namely TLE1, may be useful in this context. 

## Acknowledgments 

The authors would like to thank Samira Alminawi for her technical assistance and also Dr. Brendan Dickson, Mt Sinai Hospital, University of Toronto, Ontario, Canada. 

**Table 1:** Antibodies used for immunohistochemistry.

Antibody	Vendor	Dilution
SOX10	Santacruz (sc-365692)	1/400
BAF47	BD Biosciences	1/100
lmwCK (CAM 5.2)	Becton, Dickinson and Company	1/25
S100, panCK, CK7, CK19, EMA, bcl2, CD34, CD99, NF and Ki67	Ventana	prediluted

**Table 2:** Clinical parameters of synovial sarcoma patients.

Case#	Age, gender	location	Imaging	Treatment	Outcome
1	47 F	brachial plexus	5 × 3 cm oval enhancing	STR (50%), other n/a	Recurrence at 7 y
2	46 F	brachial plexus	8 × 5 cm multinodular	GTR, rad, chemo	Recurrence at 1 y
3	43 F	right femoral nerve	7 × 5 cm	STR, rad	Well at 9 y
4	46 F	met to T12 nerve root, abdominal wall primary	5 × 6 cm	GTR, rad, chemo	mets to lung
5	49 F	left calf	n/a	above knee amputation	mets to lung
6	74 M	met to T6 vertebrae, calf primary	n/a	GTR, chemo, rad	mets to lung, liver, vertebrae
7	59 M	C1-C5 paraspinal	6.5 × 5.5 solid & cystic	GTR, rad, chemo	Recurrence at 4 y,5 y
8	47 F	right femoral nerve	n/a	STR, rad	Well at 6 months
9	43 F	right proximal ulnar nerve	2 × 2 cm solid enhancing	GTR, rad	Recurrence at 1 y, well 13 y later

F = female; M = male; STR = subtotal resection; GTR = gross total resection; rad = radiation; chemo = chemotherapy; y = years; met = metastasis; n/a = not available.

**Table 3:** Histologic features of synovial sarcoma cases affecting the nervous system.

Case#	mono/ biphasic	growth pattern	nuclear features	staghorn vessels	collagen	stromal Ca^2+^	mitoses/ 10 hpf	molecular t(X,18)
1	mono	fascicular, focally myxoid	oval to spindled	+	thick bands	+	2	+ SYT-SSX1
2	mono	fascicular, focally myxoid	oval, small nucleoli	+	wiry	–	4	+ SYT-SSX1
3	mono	fascicular, hyalinized, focally myxoid	oval, small nucleoli	+	focally wiry	–	1	+ SYT-SSX1
4	bi (75% glandular)	large irregular glands & papillae, focal lobules & fascicles	oval, small nucleoli	–	n/a	–	23	n/a
5	bi (90% glandular)	glands of varying sizes, hyalinized & focal myxoid background	oval, small nucleoli	–	focally wiry & thick	–	18	+ SYT-SSX1
6	mono	fascicular, focal myxoid & cytoplasmic clearing	oval to round	+	focally wiry	–	30	+ SYT-SSX1
7	bi (50% glandular)	large lobules & glands, sheets & fascicles	oval to round, small nucleoli	–	focally wiry	–	2	+ SYT-SSX1
8	mono	fascicular, focally myxoid	oval, overlapping	+	focally wiry, thick	–	1	+SYT-SSX1
9	mono	fascicular, focally myxoid	oval, small nucleoli	+	–	–	22	+SYT-SSX (type n/a)

**Table 4:** Immunohistochemical results for synovial sarcoma, MPNST and synovial sarcoma mimics.

Case#	SOX10	BAF47	lmwCK	panCK	CK7	CK19	EMA	S100	CD34	bcl2	CD99	NF
Synovial sarcoma cases												
1	0	not scored	0	0	0	0	++	focal+	0	+++	+++	0
2	0	reduced	0	rare cells+	rare cells +	rare cells +	rare cells +	0	0	+++	+	0
3	0	reduced	0	+	rare cells +	+	++	0	0	++	++	0
4	0	reduced	g +++	g+++	g +++	g +++	g +++	0	0	s +++	s ++	0
5	0	reduced	g +++	g +++	g +++	g +++	g +++	0	0	s +++	s ++	0
6	0	reduced	0	0	0	0	rare cells +	0	0	++	+++	0
7	n/a	n/a	g +++	g+++	g+++	g +++	g+++	0	0	s +++	+++	0
8	0	n/a	0	0	0	0	0	0	0	++	++	0
9	0	n/a	0	rare cells+	rare cells +	0	rare cells +	0	n/a	n/a	n/a	0
MPNST cases												
10	+++	same as endothelia	0	0	rare cells +	0	0	+++	0	+	++	n/a
11*	0	reduced	0	+	0	0	+	+++	+	++	++	n/a
12	+	reduced, variable	0	0	0	0	0	+	+	+	+++	n/a
13	++	n/a	++	0	0	0	+	++	0	+	++	n/a
14	+++	same as endothelia	0	0	0	0	0	+++	0	+	+	n/a
15**	0	n/a	0	0	0	0	0	0	0	+	+	n/a
Synovial sarcoma mimics												
16 ^+^	0	same as endothelia	++	++	0	+	0	focal+	++	+	+++	+ axons
17^++^	n/a	n/a	0	0	0	0	0	0	0	++	+++	0
18^+++^	+++	same as endothelia	0	0	0	0	0	+++	0	+	++	0

*epithelioid MPNST, **SYT-SSX testing was negative; +malignant solitary fibrous tumor; ++radiation induced sarcoma (MPNST vs. fibrosarcoma); +++spindle cell melanoma; n/a = not available; g = glands; s = stroma.
